# The diagnostic value of has_circ_0006423 in non-small cell lung cancer and its role as a tumor suppressor gene that sponges miR-492

**DOI:** 10.1038/s41598-022-17816-6

**Published:** 2022-08-12

**Authors:** Linwen Zhu, Lebo Sun, Guodong Xu, Jie Song, Bingchuan Hu, Zhongjie Fang, Yanggang Dan, Ni Li, Guofeng Shao

**Affiliations:** 1 Department of Cardiothoracic Surgery, Ningbo Medical Center Lihuili Hospital, 57 Xingning Road, Yinzhou District, Ningbo City, 315040 Zhejiang China; 2 Department of Cardiothoracic Surgery, Lihuili Hospital Affiliated to Ningbo University, Ningbo City, Zhejiang China; 3 Institute of Pharmaceutics, College of Pharmaceutical Sciences, Zhejiang University, Hangzhou, Zhejiang China

**Keywords:** Genetics, Epigenetics, Gene expression, Genetic markers, Cancer, Cancer genetics, Lung cancer

## Abstract

The diagnosis and treatment of non-small cell lung cancer (NSCLC) are not ideal. We identified NSCLC-related has_circ_0006423 in database. qRT-PCR was used to measure expression levels of hsa_circ_0006423 and miR-492 in the plasma and tissue samples, and 3 NSCLC cell lines, respectively. We analyzed the relationship between expression levels of hsa_circ_0006423 and clinicopathological factors and miR-492 expression in plasma and tissue samples. Assess the diagnostic value of hsa_circ_0006423 and miR-492 in NSCLC. Cell function *vitro* experiment to explore the effect of has_circ_0006423 on NSCLC. We found has_circ_0006423 is lower expressed in NSCLC and miR-492 is opposite, has_circ_0006423 and miR-492 has diagnostic value in NSCLC. In A549 and NCI-H1299 cells, hsa_circ_0006423 inhibited the proliferation, migration, and invasion of NSCLC cells by sponging miR-492 and accelerating NSCLC cell apoptosis. This effect may be due to the combination of has_circ_0006423 and miR-492 affecting the progression of NSCLC.

## Introduction

Lung cancer is one of the most common cancers worldwide and the leading cause of cancer-related deaths. Lung cancer is one of the fastest-growing malignant tumors with the highest morbidity and mortality rates^1^. Among new cancer cases worldwide, lung cancer accounts for 12%, and 1.4 million lung cancer patients die every year^2^. Approximately 85% of lung cancers are non-small cell lung cancer (NSCLC), including lung adenocarcinoma and lung squamous cell carcinoma; about 15% of lung cancers are small cell lung cancer^3^. If NSCLC is detected early and then surgically removed, outcomes are good, and 5-year survival for small local tumors (stage I) is 70–90%. By contrast, about 75% of patients have progressed to stage III/IV or distant metastatic disease (stage IV) at the time of diagnosis. Although there have been significant advances in the treatment of advanced lung cancer, the 1-year survival rate is only 15–19%^4^. In clinical practice, diagnosis delay and lack of effective prognostic biomarkers are two primary reasons for the poor survival statistics of NSCLC patients^5^. For small cell lung cancer and NSCLC, it is essential to detect early disease, and there is an urgent need for new biomarkers to improve the accuracy of prognosis prediction, thereby improving quality of life and survival^6^.

Circular RNA (circRNA) is a non-coding RNA (ncRNA) with a covalently closed circular structure that is highly stable and conserved. Its function, mechanism, and potential diagnostic and therapeutic targets have attracted substantial attention^7^. Unlike linear RNA, the circular structure of circRNA is stable; therefore, it is not easily degraded by nucleases^8^. CircRNA was first discovered in plant viruses in 1976 and did not receive much attention initially^9^. Later, it was discovered that most circRNAs are abundant, stable, and conserved and were specific to tissues, blood samples, and developmental stages^10,11^. Several studies found that circRNA is dysregulated in tumors and participates in several biological processes of malignant tumors, including tumorigenesis, growth, invasion, metastasis, apoptosis, cycle, and angiogenesis^12,13^. Studies found that circRNA can be used as a microRNA (miRNA) sponge to regulate gene expression in various ways^14^. Several studies showed that miRNA has an essential regulatory role in tumorigenesis and progression^15–17^. These findings suggest that circRNA also participates in regulating the occurrence and progression of tumors.

At present, screening of disease-related ncRNA occurs through databases and microarrays^18,19^. In the preliminary research of this project, by screening the Gene Expression Omnibus (GEO) database from the National Center for Biotechnology Information (https://www.ncbi.nlm.nih.gov/geo/), we through GEO identified a batch of circRNA molecules that are differentially expressed in lung cancer tissues, it was found that hsa_circ_0006423 is a circRNA that is abnormally expressed in lung cancer, and there is no research related to hsa_circ_0006423 in lung cancer or other diseases after searching on PubMed^20,21^. Therefore, we chose the lung cancer-related circRNA molecule hsa_circ_0006423 from the GEO database (https://www.ncbi.nlm.nih.gov/geo/) as the object of further research. By measuring expression levels of hsa_circ_0006423 in the plasma and tissue samples of NSCLC patients, the correlation between its expression level and NSCLC was analyzed, and its feasibility as an early diagnostic marker for NSCLC was explored. Then, bioinformatics predicted the miRNA that binds to hsa_circ_0006423 (using circbank and circular RNA interactome software, miRNAs that may bind to hsa_circ_0006423 were predicted to get miR-492 after the intersection). We observed the binding between miR-492 and hsa_circ_0006423 and their role in developing NSCLC using cell experiments. Finally, we provided a basis for hsa_circ_0006423 as a diagnostic method and treatment of NSCLC.

## Materials and methods

### Patients

From September 2018 to December 2020, we enrolled 82 patients who had undergone radical NSCLC surgery in Li Huili Hospital of Ningbo Medical Center and 82 lung cancer patients who had not undergone surgery but were diagnosed with NSCLC after surgery. The diseases included lung adenocarcinoma, lung squamous cell carcinoma, and large cell carcinoma of the lung. Inclusion criteria were as follows: (1) diagnosis of NSCLC and no other malignant tumors; (2) complete medical and follow-up records; (3) fresh whole blood immediately preserved before and after surgery; and (4) age ≥ 18 years of age. Exclusion criteria were as follows: (1) metastatic lung cancer patients or lung cancer combined with other malignant tumors; (2) failure to provide complete medical and follow-up records, such as pathological records; (3) unclear pathological staging. A total of 82 healthy controls were included in the study cohort. The Medical Ethics Committee of Li Huili Hospital of Ningbo Medical Center approved the study. All human participants in this study obtained informed consent from all subjects and all studies involving human participants were conducted in accordance with the relevant provisions of the Declaration of Helsinki.

### Collection and storage of plasma and tissue

We collected 3 mL of peripheral venous blood in EDTA anticoagulation tubes, centrifuged at 3000 rpm for 15 min, removed the upper light yellow plasma layer in a 2-mL RNase-free EP tube, and stored it at -80 ℃. NSCLC tissue and matched adjacent tissues (5 cm outside the margin of the cancerous tissue) were taken from surgical patients. Tissue samples were stored immediately in RNA preservation solution (Cwbiotech, Beijing, China, cat. no. CW0592M) and stored at –80 °C until use.

### Total RNA extraction and reverse transcription

According to the manufacturer's instructions, total RNA in plasma and tissue was extracted with TRIzol LS (Invitrogen, Carlsbad, CA, USA, cat. no. 10296028) and TRIzol reagent (Invitrogen, Carlsbad, CA, USA, cat. no. 15596018), respectively. A Smart Spec Plus spectrophotometer (Denovix, Hercules, CA, USA) was used to measure the quality and concentration of total RNA. The ratio of A260/A280 absorbance was used to measure RNA quality and concentration. When the absorbance range was 1.8–2.1, the RNA quality was considered good. Reverse transcription was performed using GoScript RT Reagent (Promega, Madison, WI, USA, cat. no. A5001) or commercial miRNA reverse transcription kit (GenePharma, Shanghai, China, cat. no. E23005) to synthesize cDNA according to the manufacturer's instructions.

### qRT-PCR detects the expression level of has_circ_0006423 and miR-492

According to the kit instructions of GoTaq qPCR Reagent (Promega, Madison, WI, USA, cat. no. A6002), hsa_circ_0006423 and miR-492 were amplified on an Applied Biosystems 7500 Real-time PCR system (ThermoFisher Scientific, Rockford, IL, USA). We designed hsa_circ_0006423 specific divergent primers across the circularization site and miR-492 specific primers. Glyceraldehyde 3-phosphate dehydrogenase was used as the hsa_circ_0006423 external reference, U6 was used as the miR-492 external reference. The primer sequences are shown in Table 1. The primers were synthesized by Sangon Biotech (Shanghai, China) Co., Ltd. 2^−ΔCq^ and 2^−ΔΔCq^ were used to indicate expression level. 2^−ΔCq^ and 2^−ΔΔCq^ value correlated with expression level.

**Table 1:** Primer sequences.

Primer	Forward primer (5’ to 3’)	Reverse primer (5’ to 3’)
hsa_circ_0006423(for qPCR)	ACCTTCACCTTCAGAGTTGAGA	CCAGGGGGAACTGGTGATTC
GAPDH (for qPCR)	AAGGTGAAGGTCGGAGTCAA	AATGAAGGGGTCATTGATGG
miR-492 (for qPCR)	GGCTATGCTTGAGTACG	CTGAGTTAGCGTACGAGT
U6 (for qPCR)	GCTTCGGCAGCACATATACTAAAAT	CGCTTCACGAATTTGCGTGTCAT

### Cell culture and transfection

NSCLC cells (A549, NCI-H1299 and NCI-H1573) and human normal bronchial epithelial cells (BEAS-2B) were purchased from the Chinese Academy of Sciences Cell Bank (China). The A549 and NCI-H1299 cell lines were cultured in RPMI-1640 (HyClone, Los Angeles, CA, USA, cat. no. SH30809.01B), NCI-H1573 and BEAS-2B cell lines were cultured in Dulbecco’s modified Eagle’s medium (HyClone, Los Angeles, CA, USA, cat. no. SH30022.01B), containing 10% fetal bovine serum (Gibco, Grand Island, NY, USA, cat. no. 10100) and 1% penicillin/streptomycin (Life Technologies, Carlsbad, CA, USA, cat. no. 15140-122). The cells were grown in an incubator (Thermo Fisher, Rockford, IL, USA) containing 5% CO_2_ at 37 °C. When cell growth was in the logarithmic phase, we used the transfection reagent Lipofectamine 2000 (Invitrogen, Carlsbad, CA, USA, cat. no. 11668019) to transfect has_circ_0006423 small interfering RNA (siRNA) (GenePharma, Shanghai, China, cat. no. A01001) and siRNA control RNA (siRNA negative control [NC]), empty pcDNA3.1 vector overexpresses has_circ_0006423 recombinant plasmid and control empty pcDNA3.1 vector, miR-492 inhibitor and inhibitor NC (GenePharma, Shanghai, China, cat. no. B03001). All procedures were according to the manufacturers’ instructions.

### Fluorescence in situ hybridization (FISH)

We used Cy5 labeled specific FISH probes targeting the has_circ_0006423 and miR-492 sequence (RiboBio, Guangzhou, China). We stained cell nuclei with 4,6-diamino-2-phenyl indole (DAPI). All steps were performed per the manufacturer's instructions (RiboBio, Guangzhou, China), and the final fluorescence images were measured using a fluorescence microscope (Leica, Wetzlar, Germany).

### Dual-luciferase reporter assay

We seeded cells into 24-well plates at 1 × 10^4^ cells/well on the day before cell transfection. We then performed the experimental operation according to the manufacturer's instructions (GenePharma, Shanghai, China, cat. no. C08005), set the corresponding parameters on the fluorescence tester, measured the value of firefly luciferase activity, and finally added the stop reaction reagent to obtain renilla luciferin enzyme activity value. We then calculated changes in luciferase activity.

### Cell counting kit 8 (CCK-8) assay

For cell function experiments, we selected two NSCLC cell lines, A549 and NCI-H1299, with different metastatic abilities. The transfected A549 and NCI-H1299 cells were seeded at various time points (24 h, 47 h, 72 h, 96 h) with 5 × 10^3^ cells per well in 96-well plates and 10 μL CCK-8 reagent (Dojindo, Tokyo, Japan, cat. no. ck-04) were added to each well. Cells were incubated in 37 °C incubators for 3 h. We used a spectraMax M5 microplate reader (Molecular Devices, Silicon Valley, CA, USA) to measure the absorbance at 450 nm to estimate proliferation ability.

### Transwell migration and invasion assay

The migration and invasion ability of A549 and NCI-H1299 cells was measured using 24-well Transwell chambers (Corning, New York, NY, USA). We collected the transfected cells and resuspended them in a serum-free medium. After resuspension, 200 µL of 8 × 10^4^ cells were seeded into the upper chamber. We pre-added Matrigel (Corning, New York, NY, USA, cat. no. 356237) to the upper chamber for invasion experiments. We added 500 µL of medium containing 20% fetal bovine serum to the lower chamber. After incubating for 24 h in an incubator, cells were fixed with 4% paraformaldehyde, stained with 0.1% crystal violet, and finally counted under a microscope.

### Cell cycle assay

Cells were starved in a serum-free medium to synchronize the cell cycle. The transfected cells were collected, washed in phosphate-buffered saline (PBS), and fixed with 70% ethanol overnight in a refrigerator at –20 °C. Then, the cells were washed with pre-cooled PBS, and 1 mL PI/RNase staining buffer (Multi Sciences, Hangzhou, China, cat. no. 70-APCC101-100) was added. After staining, the cells were incubated in the dark for 30 min, and a FACS Calibur flow cytometer (Becton Dickinson Co., USA) was used to measure cell cycle steps.

### Apoptosis assay

After the cells were transfected and digested with EDTA-free trypsin, the cells were resuspended in a binding buffer. At room temperature, cells were stained using an annexin V-FITC/PI apoptosis kit (Multi Sciences, Hangzhou, China, cat. no. 70-APCC101-100) according to the manufacturer’s instructions and protected from light for 15 min. The FACS Calibur flow cytometer (Becton Dickinson Co., USA) was used to count apoptotic cells.

### Statistical analysis

We used GraphPad Prism 6.0 (GraphPad Software, San Diego, CA, USA) and Social Science Statistical Program (SPSS) 20.0 (IBM, Almont, NY, USA) software to analyze the experimental data and the data were expressed as mean ± standard deviation. The differences between groups were analyzed using two-sided Student's *t*-tests. *P* < 0.05 was considered statistically significant.

## Results

### Characterization of hsa_circ_0006423 and divergent primers

**Figure 1:**
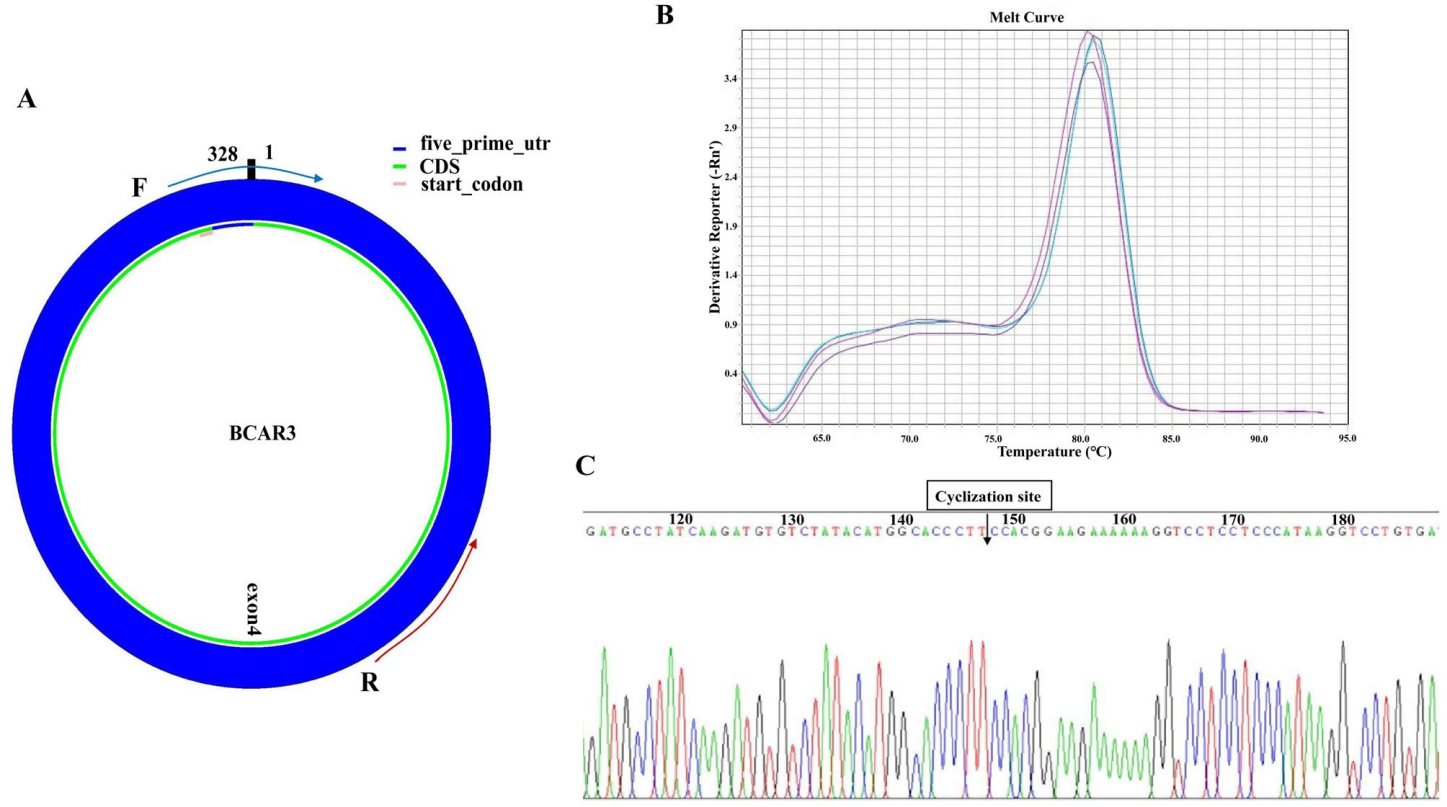
Amplification of hsa_circ_0006423. (**A**) hsa_circ_0006423 and divergent primers. Hsa_circ_0006423 comes from exon 4 of BCAR3, with a size of 328 nucleotides. The splice site sequence is in the forward primer. (**B**) Melting curves of hsa_circ_0006423 qRT-PCR products of three representative samples (the three colors represent three different samples). (**C**) Sanger sequencing results of hsa_circ_0006423. The arrow indicates the cyclization site. *BCAR3* breast cancer anti-estrogen resistance 3, *UTR* untranslated region, *CDS* coding sequence.

The hsa_circ_0006423 studied in this experiment is encoded on chromosome 1p22.1. This chromosomal region is a breast cancer anti-estrogen resistance 3 (BCAR3) mRNA transcript composed of 20 exons. The subject hsa_circ_0006423 came from exon 4 in the transcript (Fig. 1A). We designed specific hsa_circ_0006423 divergent primers to amplify hsa_circ_0006423. The melting curve in the qRT-PCR results showed that the amplified product gave a single peak (Fig. 1B), suggesting that the primers had good specificity. There was neither non-specific amplification nor primer dimer production. We also analyzed the specificity of qRT-PCR products by the Sanger sequencing method and found that the product sequence after hsa_circ_0006423 amplified by qRT-PCR contained a circularization site (Fig. 1C). This sequence is consistent with the sequence of hsa_circ_0006423 at the circBase website (http://circrna.org/). The length of the qRT-PCR product was 89 nt. These findings suggest that the primer was a specific divergent primer of hsa_circ_0006423.

### Positioning and sponge function of hsa_circ_0006423

**Figure 2:**
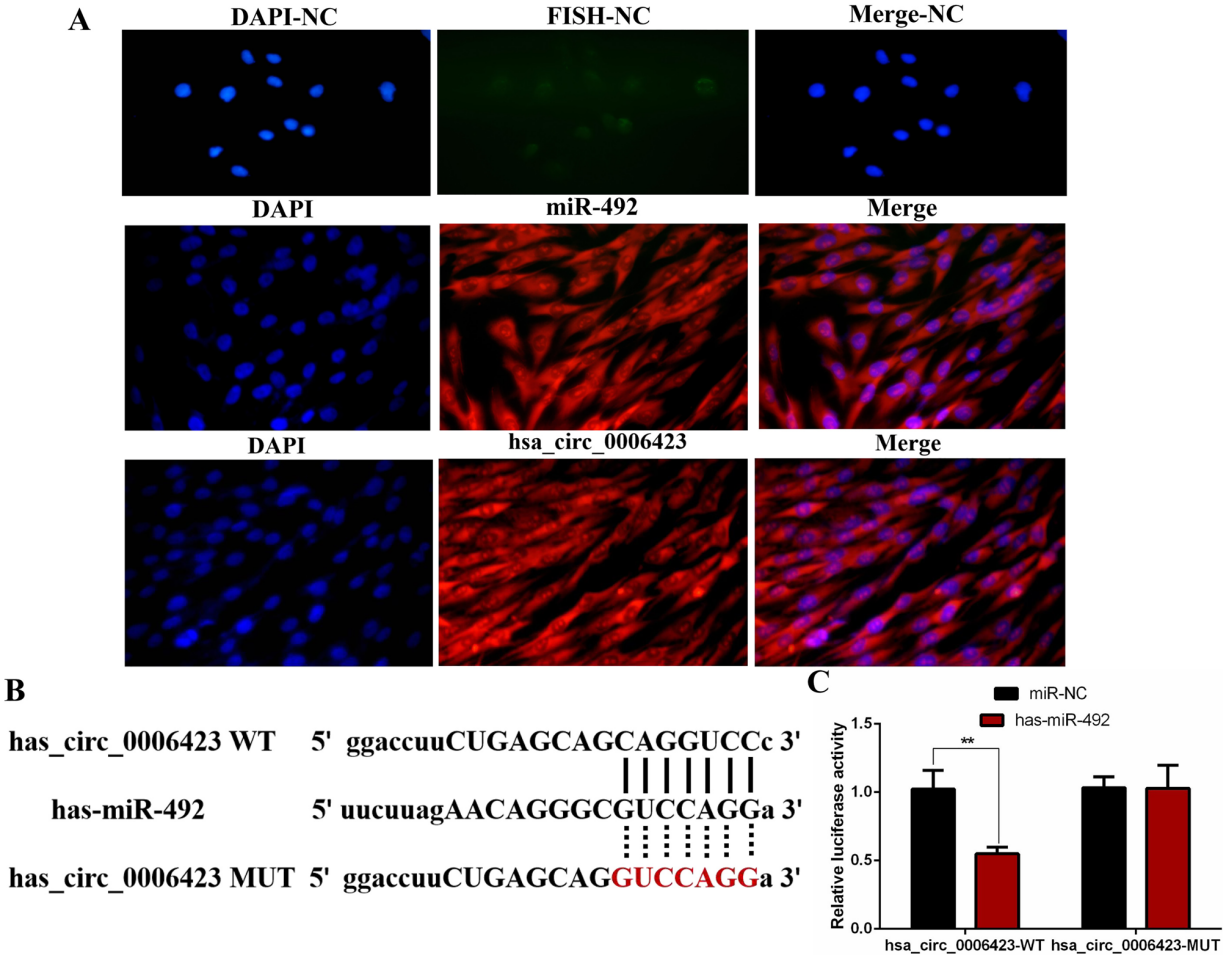
Positioning and sponge of hsa_circ_0006423. (**A**) FISH experiments confirmed that hsa_circ_0006423 and miR-492 are located in the cytoplasm. The nucleus is labeled with DAPI, hsa_circ_0006423 and miR-492 are labeled with Cy3. Three sets of repeated experiments. (**B**) The potential binding sites of hsa_circ_0006423 and miR-492. (**C**) The luciferase reporter gene experiment confirmed the combination of miR-492 and wild-type hsa_circ_0006423. *NC* negative control. **P < 0.01.

After exploring the location of hsa_circ_0000437 and miR-492 in NSCLC cell using FISH experiments, we found that hsa_circ_0006423 and miR-492 (red fluorescence) mainly concentrated in the cytoplasm (Fig. 2A). We also used Circbank and Circular RNA Interaction software to predict the miRNA that binds to hsa_circ_0006423 and get miR-492 after the intersection (Fig. 2B). MiR-492 co-localizes with hsa_circ_0006423 in the cytoplasm and might act as a sponge. We further verified the binding of hsa_circ_0006423 and miR-492 using a dual-luciferase reporter gene assay and found that hsa_circ_0006423 binds to miR-492 (Fig. 2C).

### Detect the expression level of hsa_circ_0006423 and miR-492 in plasma, tissue samples, and NSCLC cell lines

**Figure 3:**
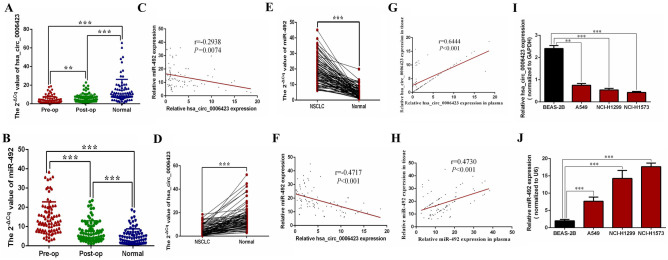
Expression levels of hsa_circ_0006423 and miR-492 in NSCLC plasma, tissue samples, and cell lines. (**A**) Expression levels of hsa_circ_0006423 in the preoperative group were significantly lower than those in the postoperative and the healthy controls group (*n* = 82), and expression levels of hsa_circ_0006423 in the postoperative group were significantly lower than those in the healthy control group (*n* = 82). (**B**) The expression levels of miR-492 in the preoperative group were significantly higher than those in the postoperative and the healthy controls group (*n* = 82), and the expression levels of miR-492 in the postoperative group were significantly higher than those in the postoperative group the healthy control group (*n* = 82). (**C**) Pearson correlation analysis revealed that expression levels of hsa_circ_0006423 and miR-492 in 82 NSCLC plasma samples were negatively correlated. (**D**) Expression levels of hsa_circ_0006423 in NSCLC tissues were significantly lower than adjacent tissues (*n* = 82). (**E**) Expression levels of miR-492 in NSCLC tissues were significantly higher than adjacent tissues (*n* = 82). (**F**) Pearson correlation analysis found that the expressions of hsa_circ_0006423 and miR-492 in 82 NSCLC tissue samples were negatively correlated. (**G**) Pearson correlation analysis found that the expressions of hsa_circ_0006423 in 82 NSCLC plasma and tissue samples were postively correlated. (**H**) Pearson correlation analysis found that the expressions of miR-492 in 82 NSCLC plasma and tissue samples were postively correlated. (**I**) Hsa_circ_0006423 in NSCLC cell lines (A549, NCI-H1299 and NCI-H1573) lower expression than the normal bronchial epithelial cell line BEAS-2B. (**J**) miR-492 in NSCLC cell lines (A549, NCI-H1299 and NCI-H1573) showed higher expression than normal bronchial epithelial cell line BEAS-2B. **P < 0.01; ***P < 0.001.

qRT-PCR was used to measure the expression of hsa_circ_0006423 and miR-492 in the preoperative, postoperative, and healthy control plasma samples of 82 NSCLC patients. We found that the preoperative and postoperative hsa_circ_0006423 expression levels in NSCLC patients were significantly lower than those of the healthy control group (*P* < 0.001; Fig. 3A); we also found that preoperative hsa_circ_0006423 levels in NSCLC patients were significantly lower than postoperative levels (*P* < 0.01; Fig. 3A). In contrast, the preoperative and postoperative miR-492 expression levels in NSCLC patients were significantly higher than those of the healthy control group (*P* < 0.001; Fig. 3B); we also found that preoperative miR-492 levels in NSCLC patients were significantly higher than postoperative levels (*P* < 0.001; Fig. 3B). Interestingly, we found that the expression levels of hsa_circ_0006423 and miR-492 in NSCLC preoperative plasma were negatively correlated (Fig. 3C).

In NSCLC tissue samples, we found that expression levels of hsa_circ_0006423 in NSCLC tissues were significantly lower than the corresponding adjacent tissues (*P* < 0.001; Fig. 3D). However, the expression levels of miR-492 in NSCLC tissues were significantly higher than the corresponding adjacent tissues (*P* < 0.001; Fig. 3E). Similarly, correlation analysis revealed that expression levels of hsa_circ_0006423 and miR-492 in NSCLC tissues were negatively correlated (Fig. 3F). Besides, we analysis the correlation between the level of hsa_circ_0006423 in preoperative plasma samples and those in tissues, found that they were positively correlated (Fig. 3G); interestingly, correlation is the same in miR-492 (Fig. 3H).

In NSCLC cell lines, we found that expression levels of hsa_circ_0006423 among 3 NSCLC cell lines with different metastatic abilities (epithelial NSCLC cells A549 without metastasis, NCI-H1299 with lymph node metastasis, and NCI-H1573 with the strongest tissue metastasis ability) were lower than that of normal bronchial cells, and the stronger the metastatic ability, the more significant the difference (Fig. 3I). Conversely, the expression level of miR-492 was higher, and it is also proportional to the metastatic abilities (Fig. 3J).

### The relationship between the expression level of hsa_circ_0006423 and clinicopathological factors

The relationship between expression levels of hsa_circ_0006423 and clinicopathological factors was analyzed based on the expression of hsa_circ_0006423 in preoperative patients in NSCLC combined with the collected clinical-pathological data. We separately analyzed the relationship between the expression of hsa_circ_0006423 and clinicopathological factors (i.e., gender, age, smoking history, histology, tumor location, differentiation, tumor size, TNM stage, driver gene, epidermal growth factor receptor [EGFR], anaplastic lymphoma kinase [ALK] and programmed cell death-ligand 1 [PD-L1]) of patients with NSCLC before surgery. We found that plasma levels of hsa_circ_0006423 in patients with NSCLC before surgery were not significantly related to clinicopathological factors (i.e., gender, age, smoking history, histology, tumor location, tumor size, TNM stage, EGFR, ALK, and PD-L1) but was related to the degree of differentiation (*P* < 0.001; Table 2); otherwise, tissue levels of hsa_circ_0006423 in patients with NSCLC before surgery was related to the tumor size (*P* = 0.002; Table 2) and PD-L1 (*P* = 0.043; Table 2). This finding suggests that higher expression levels of hsa_circ_0006423 correlate with a higher degree of differentiation of NSCLC, smaller tumor size, and lower PD-L1 positivity rate.

**Table 2:** The relationship of hsa_circ_0006423 expression levels (ΔCq) in NSCLC plasmas and tissues with clinicopathological factors of patients with NSCLC.

Characteristics	*n*	hsa_circ_0006423 level plasma (tissue)		χ^2^ test	*P* value
		High	Low		
Total cases	82	41 (41)	41 (41)		
**Gender**				0.467 (0.052)	0.495 (0.820)
Male	51	27 (25)	24 (26)		
Female	31	14 (16)	17 (15)		
**Age (years)**				0.064 (0.071)	0.800 (0.801)
≤ 60	21	10 (11)	11 (10)		
> 60	61	31 (30)	30 (31)		
**Smoking history**				0.507 (1.268)	0.821 (0.260)
Yes	33	17 (14)	16 (19)		
No	49	24 (27)	25 (22)		
**Histology**				0.830 (0.208)	0.660 (0.649)
Adenocarcinoma	38	20 (19)	18 (19)		
Squamous carcinoma	31	16 (17)	15 (14)		
Large cell lung cancer	13	5 (5)	8 (8)		
**Tumor location**				0.488 (2.390)	0.825 (0.122)
Left	41	20 (17)	21 (24)		
Right	41	21 (24)	20 (17)		
**Differentiation**				12.95 (0.518)	**0.0003** (0.472)
High and moderate	57	36 (27)	21 (30)		
Poor	25	5 (14)	20 (11)		
**Tumor size (cm)**				0.734 (9.873)	0.392 (**0.002**)
≤ 3	67	32 (28)	35 (39)		
> 3	15	9 (13)	6 (2)		
**TNM stage**				0.051 (0.456)	0.822 (0.499)
I + II	33	17 (15)	16 (18)		
III + IV	49	24 (26)	25 (23)		
**EGFR**				0.518 (0.058)	0.472 (0.810)
Positive	25	14 (13)	11 (12)		
Negative	57	27 (28)	30 (29)		
**ALK**				0.576 (0.064)	0.448 (0.800)
Positive	21	12 (10)	9 (11)		
Negative	61	29 (31)	32 (30)		
**PD-L1**				1.822 (4.100)	0.177 (**0.043**)
Positive	72	34 (33)	38 (39)		
Negative	10	7 (8)	3 (2)		

*EGFR* epidermal growth factor receptor, *ALK* anaplastic lymphoma kinase, *PD-L* programmed cell death-Ligand 1.

Significant values are in bold.

### Analysis of the diagnostic value of hsa_circ_0006423 and miR-492 in NSCLC

**Figure 4:**
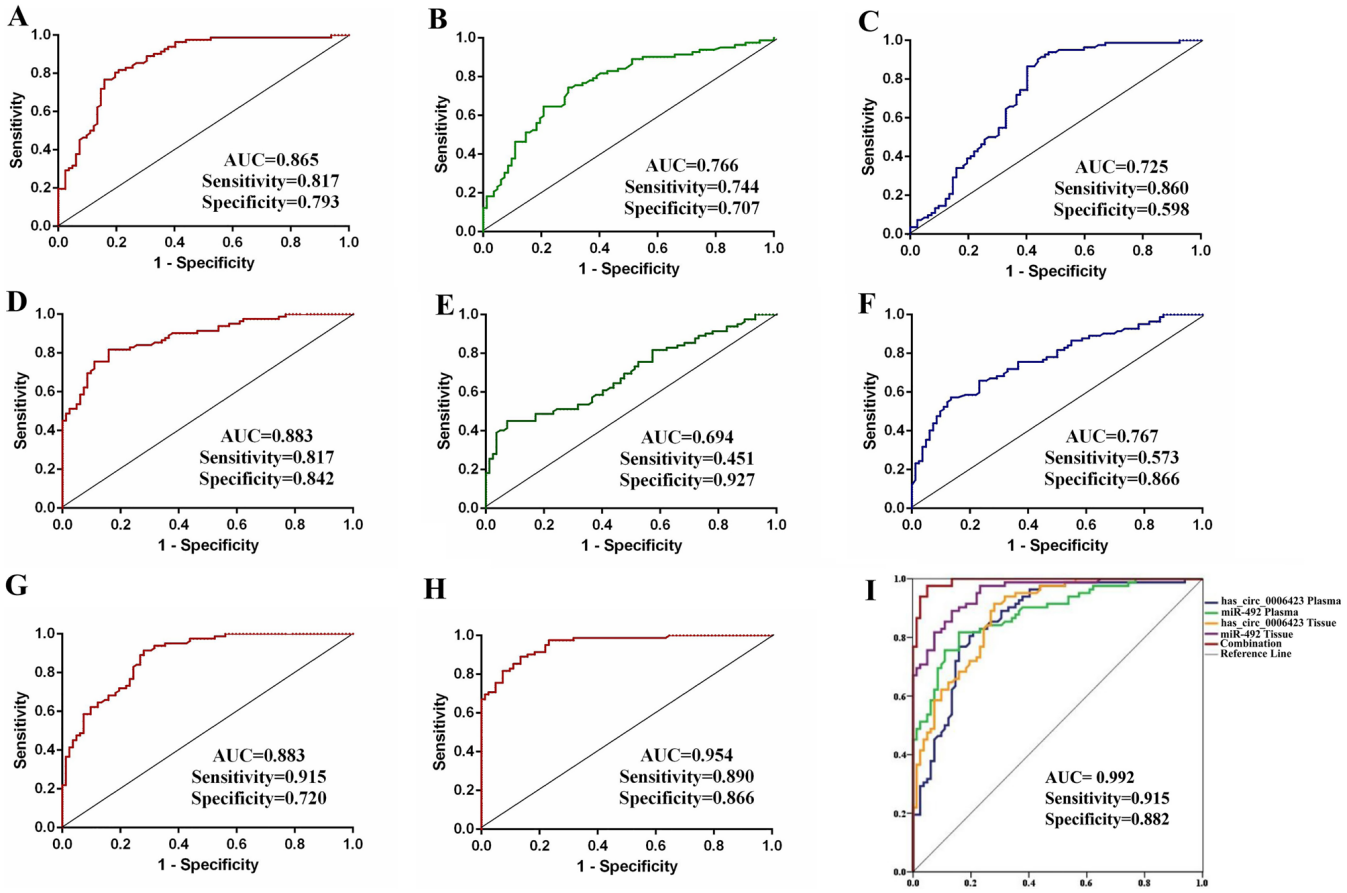
The diagnostic value of hsa_circ_0006423 and miR-492 in NSCLC. (**A**) AUC of hsa_circ_0006423 preoperative and healthy control groups in plasma samples. (**B**) AUC of hsa_circ_0006423 postoperative and healthy controls groups in plasma samples. (**C**) AUC of hsa_circ_0006423 preoperative and postoperation groups in plasma samples. (**D**) AUC of miR-492 preoperative and healthy control groups in tissue samples. (**E**) AUC of miR-492 postoperative and healthy controls groups in plasma samples. (**F**) AUC of miR-492 preoperative and postoperation groups in plasma samples. (**G**) AUC of hsa_circ_0006423 in tissue samples. (**H**) AUC of miR-492 in tissue samples. (**I**) Combined analysis of the AUC of hsa_circ_0006423 and miR-492 in plasma and tissue samples.

We analyzed the diagnostic value of hsa_circ_0006423 and miR-492 in the plasma of 82 patients with NSCLC before and after surgery and normal healthy controls. The diagnostic value was inferred by constructing ROC curves based on the area under the curve (AUC). We made ROC curves of the preoperative group of NSCLC patients to compare with the normal control group, the postoperative group of NSCLC patients compared with the normal control group, and the preoperative group of NSCLC patients compared with the postoperative group of NSCLC patients. The AUC of the preoperative group of NSCLC patients compared with the normal control group were 0.865 and 0.883, the sensitivities were 0.817 and 0.817, and the specificities were 0.793 and 0.842 in hsa_circ_0006423 and miR-492, respectively (Fig. 4A, D). Compared with the normal control group, the AUC of the postoperative NSCLC patient group were 0.766 and 0.694, the sensitivities were 0.744 and 0.451, and the specificities were 0.707 and 0.927 in hsa_circ_0006423 and miR-492, respectively (Fig. 4B, E). Compared with the postoperative group of NSCLC patients, the AUCs of the preoperative group were 0.725 and 0.767, the sensitivities were 0.860 and 0.573, and the specificities were 0.598 and 0.866 in hsa_circ_0006423 and miR-492, respectively (Fig. 4C, F). The AUCs in NSCLC tissues and adjacent tissues were 0.883 and 0.954, the sensitivity were 0.915 and 0.890, and the specificities were 0.720 and 0.866 in hsa_circ_0006423 and miR-492, respectively (Fig. 4G, H). Finally, we combined and analyzed AUC in the preoperative NSCLC patients’ compare with the normal control group, and in NSCLC tissues and adjacent tissues, the AUC was up to 0.992, the sensitivity was 0.915, and the specificity was 0.882 (Fig. 4I). These findings suggest that hsa_circ_0006423 and miR-492 have diagnostic value as biomarkers of NSCLC.

Traditional lung cancer diagnostic biomarkers include neuron-specific enolase, cytokeratin-19-fragment, and cancer antigen 72–4. We found that hsa_circ_0006423 and miR-492 had a better diagnostic value as a non-invasive biomarker in NSCLC than these traditional markers (Table 3).

**Table 3:** Diagnostic values of has_circ_0006423, miR-492 and 3 traditional lung cancer markers.

Item	AUC	Sensitivity	Specificity
Pre-op versus Healthy (hsa_circ_0006423)	0.865	0.817	0.793
Pre-op versus Healthy (miR-492)	0.883	0.817	0.842
Post-op versus Healthy (hsa_circ_0006423)	0.766	0.744	0.707
Post-op versus Healthy (miR-492)	0.694	0.451	0.927
Pre-op versus Post-op (hsa_circ_0006423)	0.725	0.860	0.598
Pre-op versus Post-op (miR-492)	0.767	0.573	0.866
Cancer versus Adjacent (hsa_circ_0006423)	0.883	0.915	0.720
Cancer versus Adjacent (miR-492)	0.954	0.890	0.866
Combination	0.992	0.915	0.882
NSE	0.619	0.733	0.567
CYFR21-1	0.586	0.633	0.633
CA72-4	0.521	0.867	0.367

*NSE* neuron-specific enolase, *CYFR21-1* cytokeratin-19-fragment, *CA72-4* cancer antigen 72-4.

### Hsa_circ_0006423 inhibits the proliferation of NSCLC cells

**Figure 5:**
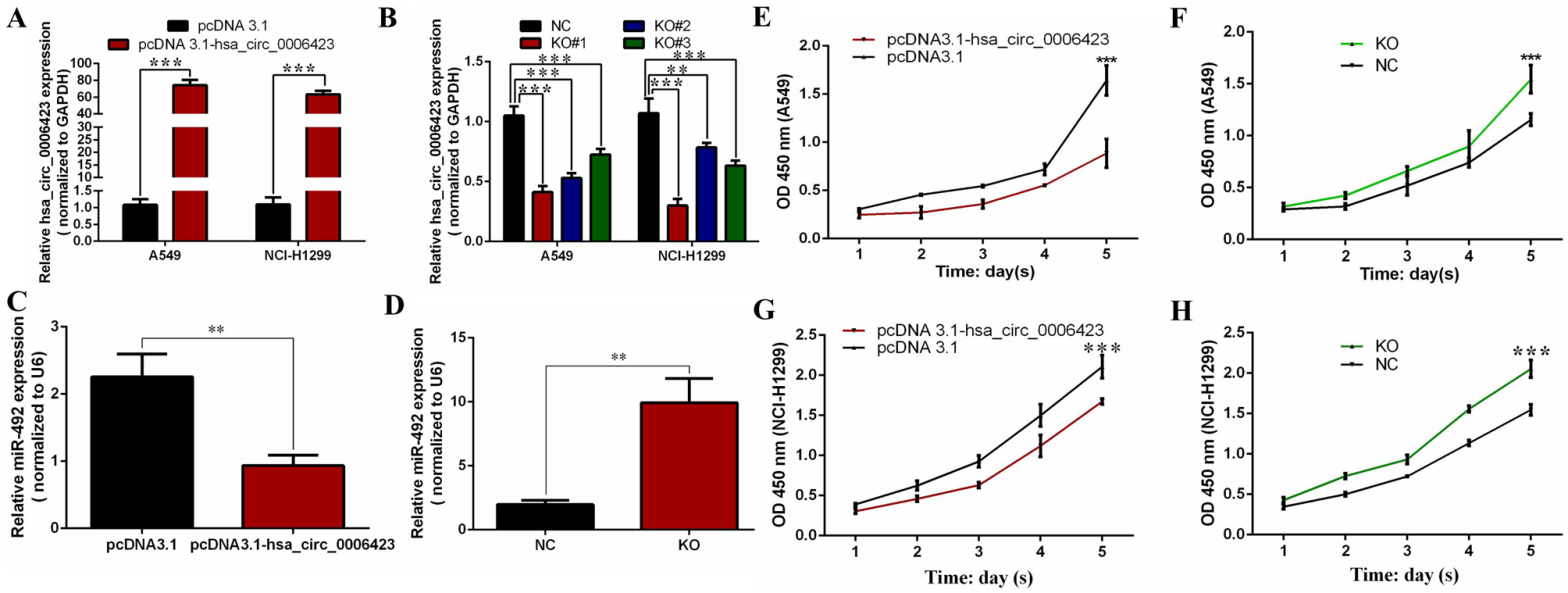
Expression levels of hsa_circ_0006423 after upregulation and downregulation of hsa_circ_0006423; effects of hsa_circ_0006423 on NSCLC proliferation. (**A**) Upregulation effect of hsa_circ_0006423 overexpression plasmid in NSCLC cells. (**B**) siRNA downregulation effect of hsa_circ_0006423 in NSCLC cells. (**C**) Expression level of miR-492 before and after overexpression of hsa_circ_0006423. (**D**) Expression level of miR-492 before and after knockdown of hsa_circ_0006423. (**E**) Grow curve of A549 cells following overexpression of hsa_circ_0006423. (**F**) Grow curve of A549 cells following silencing of hsa_circ_0006423. (**G**) Growth curve of NCI-H1299 cells following overexpression of hsa_circ_0006423. (**H**) Growth curve of NCI-H1299 cells following silencing of hsa_circ_0006423. ***P < 0.001.

The hsa_circ_0006423 overexpression plasmid and blank NC vector pcDNA3.1 were transfected into the NSCLC cell lines A549 and NCI-H1299. siRNA and NC of hsa_circ_0006423 were also transfected into these cell lines and were found to upregulate and downregulate expression levels of hsa_circ_0006423, respectively (Fig. 5A, B). We chose knockout #1 (KO#1) for follow-up experiments, which had the highest downregulation efficiency. Upregulation was the only recombinant plasmid containing the full-length sequence. We found that the level of miR-492 before and after overexpression or knockdown of hsa_circ_0006423 was decreased and increased, respectively (Fig. 5C, D). Using CCK-8 experimental analysis, we found that overexpression of hsa_circ_0006423 significantly inhibited the proliferation of A549 and NCI-H1299 cells (Fig. 5E, G). When expression levels of hsa_circ_0006423 were silenced, the proliferation ability of A549 and NCI-H1299 cells significantly improved (Fig. 5F, H), suggesting that hsa_circ_0006423 inhibits the proliferation of NSCLC cells.

### Hsa_circ_0006423 inhibits migration and invasion of NSCLC cells

**Figure 6:**
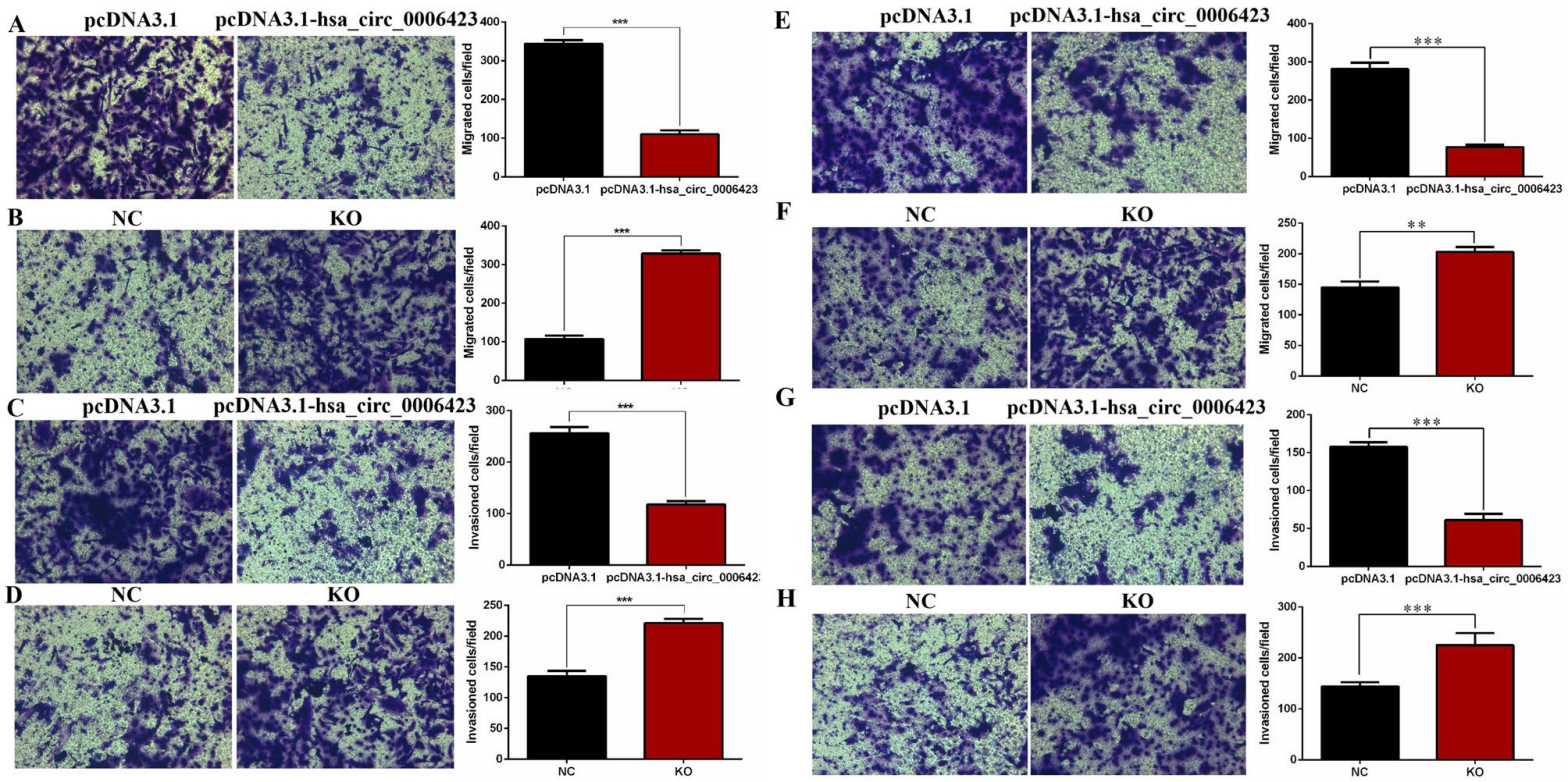
Effects of hsa_circ_0006423 on NSCLC cell lines migration and invasion. (**A**) Cell migration numbers of A549 following overexpression of hsa_circ_0006423. (**B**) Cell migration numbers of A549 following silencing of hsa_circ_0006423. (**C**) Cell invasion numbers of A549 following overexpression of hsa_circ_0006423. (**D**) Cell invasion numbers of A549 following silencing of hsa_circ_0006423. (**E**) Cell migration numbers of NCI-H1299 following overexpression of hsa_circ_0006423. (**F**) Cell migration numbers of NCI-H1299 following silencing of hsa_circ_0006423. (**G**) Cell invasion numbers of NCI-H1299 following overexpression of hsa_circ_0006423. (**H**) Cell invasion numbers of NCI-H1299 following silencing of hsa_circ_0006423. **P < 0.01; ***P < 0.001.

Transwell experiments showed that overexpression of hsa_circ_0006423 significantly inhibited the migration and invasion of A549 (Fig. 6A, C) and NCI-H1299 (Fig. 6E, G) cells. When hsa_circ_0006423 was silenced, the migration and invasion of A549 (Fig. 6B, D) and NCI-H1299 (Fig. 6F, H) cells significantly increased, suggesting that hsa_circ_0006423 inhibits the migration and invasion of NSCLC cells.

### Hsa_circ_0006423 affects cell cycle progression and promotes apoptosis of NSCLC cells

**Figure 7:**
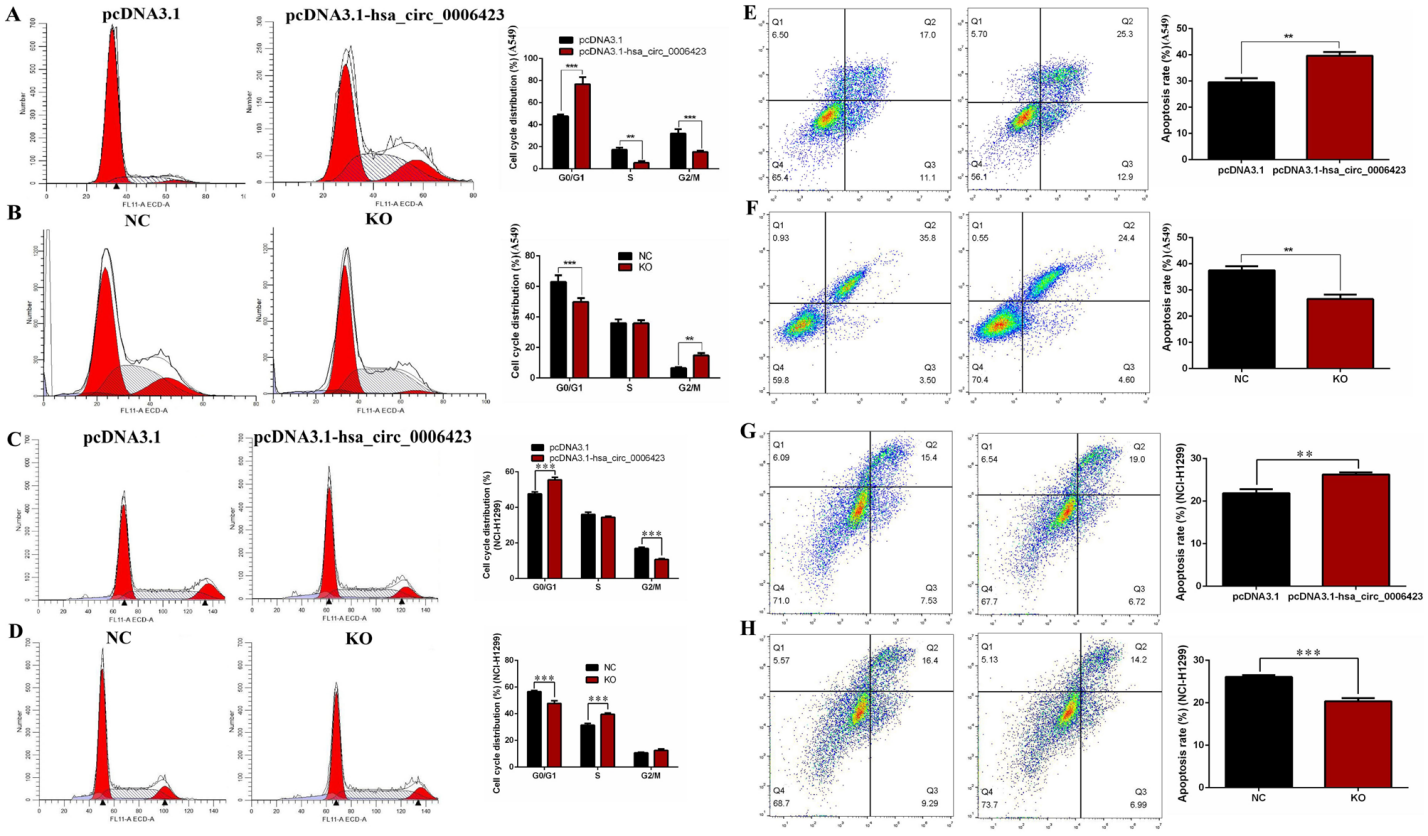
Effects of hsa_circ_0006423 on NSCLC cell line cell cycle distribution and apoptosis. (**A**) Cell cycle distribution of A549 following overexpression of hsa_circ_0006423. (**B**) Cell cycle distribution of A549 following silencing of hsa_circ_0006423. (**C**) Cell cycle distribution of NCI-H1299 following overexpression of hsa_circ_0006423. (**D**) Cell cycle distribution of NCI-H1299 following silencing of hsa_circ_0006423. (**E**) The apoptosis rate of A549 following overexpression of hsa_circ_0006423. (**F**) The apoptosis rate of A549 following silencing of hsa_circ_0006423. (**G**) The apoptosis rate of NCI-H1299 following overexpression of hsa_circ_0006423. (**H**) The apoptosis rate of NCI-H1299 following silencing of hsa_circ_0006423. **P < 0.01; ***P < 0.001.

Using flow cytometry experimental analysis, we found that overexpression of hsa_circ_0006423 blocked the cell cycle in the G0/G1 phase (Fig. 7A, C). When hsa_circ_0006423 was silenced, the cell cycle was blocked in the S and G2/M phases (Fig. 7B, D).

Using flow cytometry, we also found that overexpression of hsa_circ_0006423 significantly increased apoptosis (Fig. 7E, G). When hsa_circ_0006423 was silenced, the number of apoptotic cells was significantly reduced (Fig. 7F, H), suggesting that hsa_circ_0006423 promotes apoptosis in NSCLC cells.

### Has_circ_0006423 inhibits the proliferation, migration, and invasion of NSCLC cells by regulating miR-492

**Figure 8:**
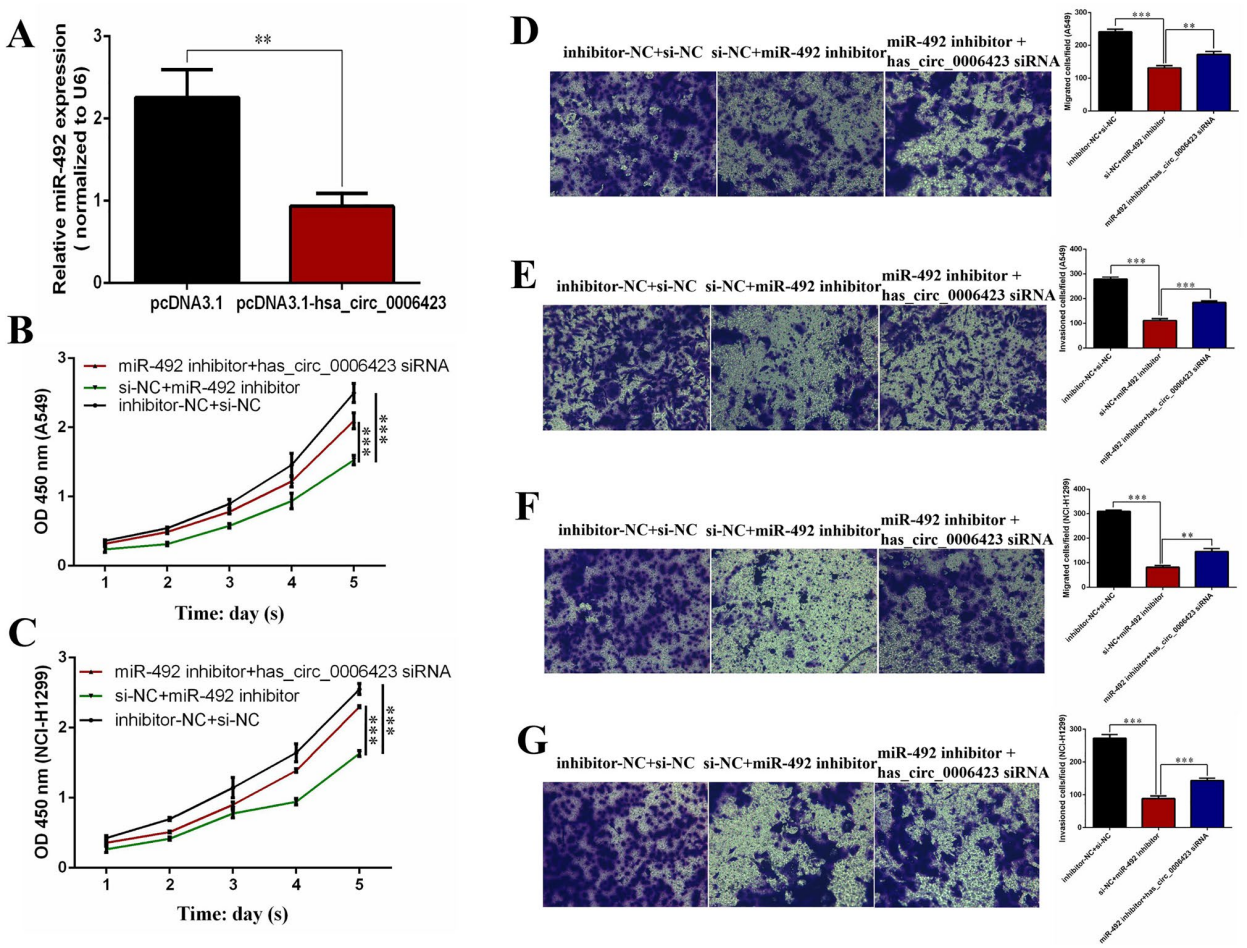
hsa_circ_0006423 inhibits the proliferation, metastasis, and invasion of NSCLC cells by inhibiting miR-492. (**A**) Expression level of miR-492 after transfecting miR-492 inhibitor. (**B, C**) The rescue effect after silencing hsa_circ_0006423 and miR-492 at the same time. CCK-8 proliferation assay in A549 and NCI-H1299 cells. (**D, E**) The rescue effect after silencing hsa_circ_0006423 and miR-492 at the same time. Transwell migration and invasion assay in A549 cells. (**F, G**) The rescue effect after silencing hsa_circ_0006423 and miR-492 at the same time. Transwell migration and invasion assays in NCI-H1299 cells. **P < 0.01; ***P < 0.001.

To determine whether has_circ_0006423 affects phenotypes of NSCLC cells through sponging miR-492, we used rescue experiments to measure the effects of has_circ_0006423 and miR-492 on the phenotype (proliferation, migration, and invasion) of NSCLC cells A549 and NCI-H1299. First, we found that the expression level of miR-492 could be significantly inhibited by transfecting miR-492 inhibitor (Fig. 8A). We found that silencing has_circ_0006423 in A549 (Fig. 8B) and NCI-H1299 (Fig. 8C) cells alleviated the cell growth inhibition induced by miR-492 silencing. Similarly, treatment with has_circ_0006423 siRNA in the two NCLC cell lines restored the reduction in migration (Fig. 8D, F) and invasion (Fig. 8E, G) caused by miR-492 silencing. These findings suggest that has_circ_0006423 regulates the phenotype of NSCLC cells through sponge adsorption of miR-492, thereby inhibiting cell proliferation, migration, and invasion.

## Discussion

Although some progress has been made in diagnosing and treating lung cancer, the disease remains a substantial problem worldwide^22^. Compared with other malignant tumors, lung cancer imposes greater financial burdens on families^23^. There are no clinically specific and sensitive diagnostic tools to distinguish lung cancer patients from healthy people or track outcomes. Effective treatments for lung cancer also need to be improved. Therefore, it is imperative to identify early detection biomarkers with prognostic potential and identify effective therapeutic targets^24,25^. CircRNA represents a class of conserved, endogenous RNA that regulates mammalian gene expression^26^. CircRNA is abundant in human body fluids and can regulate the tumor microenvironment via cell-to-cell communication^27^. Unlike non-coding RNAs such as miRNAs and long non-coding RNAs, circRNAs have a high degree of sequence conservation and stability in mammalian cells^26,28,29^. It is these characteristics that provide circRNAs with the potential to become ideal biomarkers and potential therapeutic targets. Zhu et al. found a correlation between the upregulation of circRNA 100,876 and NSCLC lymph node metastasis^30^, hsa_circ_0013958 is thought to be a miR-134 sponge that gives rise to the upregulation of cancer-causing cyclin D1 in lung adenocarcinoma^31^. For the sponge miR-492 of hsa_circ_0006423 in this study, it was found that miR-492 was highly expressed in NSCLC serum^32^; and silencing miR-492 inhibited the proliferation, migration and invasion of NSCLC cells^33^. This provides a basis for further research on the inhibition of NSCLC occurrence and development after the hsa_circ_0006423 sponge absorbs miR-492. Although many researchers are beginning to study the potential functions of circRNA, their clinical diagnostic and therapeutic value remains largely unknown.

Although thousands of circRNAs have been identified in lung cancer tissues and cell lines using high-throughput sequencing technology, and many circRNAs are abnormally expressed in lung cancer, research concerning their specific functions and mechanisms in the occurrence and progression of lung cancer has only recently begun^34^. We detected the hsa_circ_0006423 level in plasmas, tissues, and cells of NSCLC for the first time. Plasma expression levels of hsa_circ_0006423 in NSCLC plasmas, tissues, and cells were significantly lower than those of healthy controls, suggesting preliminarily that hsa_circ_0006423 can be used as a biomarker for the diagnosis of NSCLC. Combined with clinicopathological data, we found a positive correlation with degree of differentiation in plasma samples and a negative correlation with tumor size and PD-L1 expression in tissue samples, this difference in clinicopathological associations may be due to the limited sample size. The correlation between the expression of has_circ_0006423 and PD-L1 in tissue samples revealed that the immune environment is correlated with the expression level of has_circ_0006423, which may provide a potential immunotherapy target for the diagnosis and treatment of NSCLC. Besides, lower expression levels of hsa_circ_0006423 indicated a worse pathological type, these findings suggest that hsa_circ_0006423 may predict survival. AUC up to 0.992, suggesting that hsa_circ_0006423 can be used as a non-invasive diagnostic biomarker for NSCLC. We further studied the effect of hsa_circ_0006423 on the biological functions of NSCLC cells. We found that overexpression of hsa_circ_0006423 inhibited the proliferation, migration, invasion, and cell cycle progression of NSCLC cells and promoted NSCLC apoptosis while silencing hsa_circ_0006423 promoted proliferation, migration, invasion, and cell cycle progression of NSCLC cells and inhibited apoptosis. The occurrence and development of tumors are closely related to proliferation and migration. This study found that hsa_circ_0006423 and miR-492 can respectively inhibit and promote the proliferation and migration of NSCLC cells, thereby inhibiting and promoting the occurrence and development of NSCLC. These findings provide a basis for using hsa_circ_0006423 as a tumor suppressor in NSCLC. Co-localization of hsa_circ_0006423 and miR-492 in the cytoplasm might provide a basis for targeted therapy of NSCLC.

In summary, our findings suggest that hsa_circ_0006423 is involved in the carcinogenesis and progression of NSCLC, may serve as a potential therapeutic target for NSCLC, and may serve as a potential non-invasive blood biomarker for early detection of NSCLC. Further clinical trials and ethical approvals are needed before use in patients. There remains a lack of research on the mechanisms of inhibition of the progression of NSCLC after has_circ_0006423 sponges miR-492. Has_circ_0006423 may affect the development of NSCLC by regulating or targeting binding proteins or mRNA. These mechanisms need to be studied further. It is also necessary to validate our findings using animal experiments.

## Data Availability

Data sets related to this article are available at GEO resource (https://www.ncbi.nlm.nih.gov/geo/).
